# Primary Repair of Moderate Severity Rhegmatogenous Retinal Detachment: A Critical Decision-Making Algorithm

**Published:** 2016

**Authors:** Raul VELEZ-MONTOYA, Paola JACOBO-OCEGUERA, Javier FLORES-PRECIADO, Jose DALMA-WEISZHAUSZ, Jose GUERRERO-NARANJO, Guillermo SALCEDO-VILLANUEVA, Gerardo GARCIA-AGUIRRE, Jans FROMOW-GUERRA, Virgilio MORALES-CANTON

**Affiliations:** 1 Retina Department. Asociación para Evitar la Ceguera en México IAP, México City DF, Mexico

**Keywords:** Primary Repair, Rhegmatogenous Retinal Detachment, Algorithm, Scleral Buckle, Primary Pars Plana Vitrectomy

## Abstract

We reviewed all the available data regarding the current management of non-complex rhegmatogenous retinal detachment and aimed to propose a new decision-making algorithm aimed to improve the single surgery success rate for mid-severity rhegmatogenous retinal detachment. An online review of the Pubmed database was performed. We searched for all available manuscripts about the anatomical and functional outcomes after the surgical management, by either scleral buckle or primary pars plana vitrectomy, of retinal detachment. The search was limited to articles published from January 1995 to December 2015. All articles obtained from the search were carefully screened and their references were manually reviewed for additional relevant data. Our search specifically focused on preoperative clinical data that were associated with the surgical outcomes. After categorizing the available data according to their level of evidence, with randomized-controlled clinical trials as the highest possible level of evidence, followed by retrospective studies, and retrospective case series as the lowest level of evidence, we proceeded to design a logical decision-making algorithm, enhanced by our experiences as retinal surgeons. A total of 7 randomized-controlled clinical trials, 19 retrospective studies, and 9 case series were considered. Additional articles were also included in order to support the observations further. Rhegmatogenous retinal detachment is a potentially blinding disorder. Its surgical management seems to depend more on a surgeon´s preference than solid scientific data or is based on a good clinical history and examination. The algorithms proposed herein strive to offer a more rational approach to improve both anatomical and functional outcomes after the first surgery.

## INTRODUCION

Rhegmatogenous retinal detachment (RRD) is defined as the separation of the neuroretina from the retinal pigment epithelium, secondary to the passage of liquefied vitreous into the subretinal space, through a hole or tear in the neuroretina, regardless of its localization (1-3). It is the most common form of retinal detachment and constitutes a disease with a high risk of severe visual impairment and complications, such as hypotony and phthisis, if left untreated (4, 5). The clinical presentation varies widely and can range from relatively uncomplicated, with a single break and localized detachment, to multiple, large, odd shaped breaks, with total detachment and preoperative proliferative vitreoretinopathy (PVR) (6). The annual reported incidence of RRD ranges from 7 to 13 cases per 100,000 people (4, 7). However, the number of cases seems to be trending upward, most likely due to the longer life span of the general population and the increasing popularity of anterior segment surgeries like cataract extraction (overall cumulative RRD after phacoemulsification of 0.39-1.0% over a follow up of ≈5 years) (3, 8) (9, 10).

The treatment of RRD is one of the most frequent indications for vitreoretinal surgery (21,762 repairs in 2009 according to Medicare database) and constitutes about half of all surgical cases in busy vitreoretinal practices (5, 6, 11). Although there is little doubt about the necessity of treatment for symptomatic RRDs since it has proven to be a sight-saving and cost-effective procedure (5, 12), there is controversy and no general consensus regarding the best surgical approach. This is especially true for mid-severity cases, such as cases with multiple, large or unusually shaped breaks, breaks posterior to the equator, or RRD in pseudophakic patients with no visible lesion. Conversely, most surgeons will agree on the method of surgical repair for cases located in the poles of the severity spectrum.(13, 14) Treatment choices have changed considerably in the recent decades. Currently, there are four main surgical techniques: pneumoretinopexy, scleral buckle (SB), primary pars plana vitrectomy (PPV), and a combination of PPV and SB (PPV/SB) (15, 16). The latest technological advances in vitrectomy machines (pumps, cutting probes, vacuum control, and adjustable duty cycles), wide-field non-contact visualization systems, as well as surgical adjuvants, such as triamcinolone, vital dyes, endolaser probes and perfluorocarbon liquids, have increased the number of cases of RRD managed with vitrectomy. Whereas the number of cases treated with SB have been reducing in the las few years (13, 16-18). Currently, in the United States (US), vitrectomy is the first choice for the treatment in more than 60% of RRDs (4, 15). A similar trend is seen in Europe, where there is a clear tendency toward choosing PPV over SB (19-21).

It seems that the surgical repair of uncomplicated RRD of mid-severity still remains a highly individualized procedure (6). The technique of choice appears to depend, more often than it should, on surgeon preferences than on preoperative findings or patient characteristics (14). Although there seems to be no difference in the rates of final re-attachment among the different techniques, an important number of clinical trials, retrospective surveys, case series, and meta-analyses have been performed in order to attempt to identify prognostic factors that will help surgeons predict the anatomical or functional outcome. However, the results have been inconsistent (16, 22-26) and difficult to apply to daily practice, mainly due to the design of the trials and the way they are conducted (6). Moreover, the variability with which the outcomes are qualified, poor follow-up, the differences among enrolled populations, lack of complete or essential reported data (such as macular status [on/off]), inadequate power and sample size, and an inconsistent definition of “success” among the studies make them very difficult to compare and draw more definite conclusions (6, 15, 27).

The following manuscript aims to filter and categorize the most relevant published data from the last decade about the anatomical and functional outcome, and how they can be affected by preoperative clinical factors, when selecting SB or PPV as the primary reattachment technique. The ultimate purpose is to try to design a logical critical decision-making algorithm with the available data, in order to maximally improve the single surgery success rate (SSSR) of uncomplicated, mildly to moderately severe RRDs, as well as the final visual outcome. SSSR is defined as the number of retinas that remain attached after the first surgical procedure, without the need for further interventions, gas, laser, or any other invasive procedure. We limited our database search to the last decade, in order to consider only studies with the latest technology in vitrectomy machines, surgical adjuvants, and small gauge vitrectomy. We included relevant randomized-controlled trials, retrospective studies, and case series with at least three months of follow-up. We categorized the level of information according to the study design of each reference. We gave the highest level of relevance to data from randomized prospective clinical trials and meta-analyses, followed by data from retrospective studies, and finally the result of well-followed case series. We do not considered this review a meta-analysis, but is a logical abstraction of the existing data with the addition of the experiences regarding the management of this type of RRD gathered from our department. Therefore, the content of this manuscript was not limited to rough data, but in addition, the authors highlight what they considered to be the most important factors or factors that might have major impact in the anatomic or functional outcome when treating these cases.

## Scleral Buckle for Uncomplicated Mid-Severity RRD

Along with pneumoretinopexy, these techniques are the undisputed managing methods for uncomplicated RRD (mild) (6, 28). These are detachments limited to a few clock hours with a single, small, anterior, and well defined retinal lesion, usually a hole, a retinal dialysis, or a tear with limited traction and no PVR (2, 3, 28). The encircling band of solid or porous silicon is used to create a scleral indentation and support equatorial or pre-equatorial breaks, to reduce tractional forces from the peripheral vitreous (14, 29). The indentation should be high enough to neutralize the vitreous traction and the break must be adequately supported on the apex of the indentation or immediately adjacent to the anterior slope to prevent reopening (2, 30). The reported anatomical success rate ranges from 63.6% to 100% depending on the reference (14, 31). In addition, the United Kingdom National Ophthalmology Database Study of Vitreoretinal Surgery published in its report #3 that SB also has a low incidence of complications (3.6%, 95% confidence interval [CI]: 2.2-5.9), low redetachment rates (12.3%), and some improvement of visual acuity in 71.6% of the cases (15). Table 1 summarizes some of the most relevant studies regarding RRD and SB.

In 2001 the Scleral Buckling versus Primary Vitrectomy in Rhegmatogenous Retinal Detachment study (SPR study) was designed as a randomized prospective study to assess the differences in the final best corrected visual acuity (BCVA), anatomical outcome, reoperation rates, and cataract formation rates between both techniques (14, 19). One of the study´s main contributions was that it proved that SB significantly decreases the risk of recurrence in phakic eyes and provides a better functional outcome (14, 19, 27, 31, 32). Similar results have been replicated in other studies (6). In the series published by Mansouri in 2010, SB lowered the requirement of a secondary procedure for retinal reattachment after the initial surgery to a greater extent than PPV (33). The conclusion is very similar to the SPR conclusion, when taking into account that 58 out of 63 enrolled patients in Mansouri’s study were phakic (33). Moreover, the Retinal Detachment Study from the European Vitreoretinal Society (EVRS) reported that in cases of uncomplicated RRD, patients who underwent SB had lower rates of detached retinas that were judged to be inoperable at the end of the study, when compared to the other two techniques (PPV and PPV/SB) (24). Again, their conclusion is also in concordance with the SPR study results, most likely because the EVRS study had almost 5 times more phakic patients enrolled than pseudo/aphakic patients (1103 vs. 238) (24).

Through the many reports of the SPR study, the group has also been able to identify factors that significantly affect the anatomical success in phakic eyes (31). Regardless of the technique, the number of retinal breaks (6 or more; OR: 0.7, 95% CI: 0.67-0.8, P < 0.001) and the break extension (> 1 clock hour; OR: 0.3, 95% CI: 0.21-0.64 P < 0.001) were negatively associated with anatomical success (27, 31, 32).

**Table 1:** Summary of the most relevant data regarding RRD and its surgical management with SB since 2009.

**Year**	**Author**	**Type of Study **	**Main Outcome**	**Number of Participants.**
2007	Heimann et al.	RCT	SB vs. PPV for RRD: Phakic better with SB, pseudophakic better with PPV.	SB: 342; PPV: 339
			Anatomical outcome: SB: Primary: 63.6%. Final: 96.7%. BCVA: +0.39logMAR (*p<0.01*).	Total: 681.
2009	Banaee et al.	RS	Outcomes of tree different SB techniques.	Phakic: 90; PseudoP: 21
			Multiple breaks = poor anatomical outcome *(p<0.01)*; preoperative macular status correlate with visual outcomes.	Total: 111.
2009	Kim et al.	RCT	Persistence of SMF after macula affecting RRD and visual outcomes.	SB: 45; PPV: 61 (86% phakic)
			55.6% in SB group had SMF after 1 months (*p<0.01*) with worst BCVA at 6 and 12 months.	Total: 106.
2010	Mansouri et al.	RS	Clinical outcomes of re-detached retinas after primary RRD repairment.	Phakic: 168; PseudoP: 118.
			Success of secondary procedure was lower in SB (phakic) than in PPV, but required less silicon and had better BCVA (20/50 or better).	Total: 286.
2010	Goezinne et al.	RS	Clinical variables associated with redetachment and poor vision.	Phakic: 339; PseudoP: 97.
			Cumulative size of lesion (+3DD) predictor for redetachment. Redetachment and more than 7 days of visual field loss had poorer vision.	Total: 436.
2011	Schaal et al.	RS	Functional and anatomic outcomes after RRD surgery.	Phakic: 175; Pseudo P: 147
			VA at 1 year was equal among all surgical techniques.	Total: 322.
2011	Heussen et al.	RCT	Risk factors that may lead to reoperations: SPF report no. 4.	Phakic: 416; PseudoP: 265.
			Recurrence significant reduced in Pseudo P with PPV and increased in phakic (*p<0.01*).	Total: 681.
			**RF Phakic** (*p<0.01*): low IOP and persistent intraoperative detachment at the buckle.	
			**RF PseudoP** (*p<0.05*): Preoperative low BCVA, YAG capsulotomy, large breaks number of affected quadrants, retinal incarceration	
			during surgery, number of breaks and symptomatic visual field defect.	
2011	Heussen et al.	RCT	Risk factors associated to better BCVA at 12 months: SPR report no. 6.	SB: 342; PPV: 339.
			**RF Phakic** (*p<0.05*): Number of breaks, duration of symptoms, baseline BCVA, retinal detachment central to major vessels arcades,	Total: 681.
			total detachment, and chain formation of breaks.	
			**RF PseudoP** (*p<0.05*): Number of breaks, secondary cataract or central capsular fibrosis, intraoperative photocoagulation and	
			inferior detachment with breaks below the 4 and 8 o’clock positions.	
2012	Thelen et al.	RS	Anatomical outcome after SB in macula on and macula off patients	RRD: 3956; Eyes: 4325.
			Success rate macula-off: 80.46% (7.78% lower; *p < 0.01*) than macula-on (88.24%)	Total 3956 (Phakic: 3151).
2013	Oluleye et al.	RS	Anatomical and visual outcomes after SB.	SB: 45
			Anatomical success rate of 95.6%. All patients were phakic.	Total: 45.
2013	Bernheim et al.	RCT	Anatomical and Functional outcomes in phakic and pseudophakic eyes with high myopia.	Phakic: 107; PseudoP: 8.
			Initial visual acuity, axial length and pars plana vitrectomy were significantly predictive of good final visual acuity	Total: 115.
2013	Huang et al.	RCS	Macular recovery by OCT for macula off RRD: SB vs. PPV.	SB: 32; PPV: 26.
			PPV was better choice for macular recovery but had higher incidence of epiretinal membranes during follow-up.	Total: 58.
2013	Adelman et al.	RCT	Surgical outcomes for complex RRD: EVRS report no. 2.	Total: 516.
			SB had higher rate of failure with PVR B. Giant tear, hypotony, choroidal detachment had higher anatomical failure with SB.	
2013	Adelman et al.	RCT	Surgical outcomes for uncomplicated RRD: EVRS report no. 1.	Phakic: 1103; PseudoP: 238.
			SB had better anatomical outcome than PPV and PPV/SB in phakic patients. There was no difference between segmental and 360 buckle.	Total: 1341.
2014	Wong et al.	RS	Trends and outcomes for RRD surgical repair in in a large Asian tertiary eye center.	Phakic: 629; PseudoP: 158.
			Better functional outcome achieved with SB than with PPV and PPB/SB (*p<0.01*). Macula on: 62.3%.	Total: 787.
2014	Kobashi et al.	RS	Anatomical outcomes of SB for RRD and prognostic factors for primary anatomical success.	SB: 271 (Phakic: 260).
			Macula-off RRD had lower primary anatomical success rates than macula-on RRD (*p<0.01*).	PPV: 271 (Phakic: 228).
			Macula status is an independent risk factor for redetachment in SB (*p<0.01*).	Total: 542.
2014	Jackson et al.	RS	Characteristics, complications and outcomes of retina surgery for RRD.	Total: 413.
			Primary anatomical outcome 87.6%. Redetachment: 12.3%. BCVA improvement in 71.6%.	
				Total: 10396 cases

Likewise, on a retrospective study by Banaee et al. in 2009 and by Goezinne et al. in 2010, worse anatomical outcomes were observed in patients with multiple breaks and a cumulative size of tears greater than 3 disk diameters (34, 35). Despite the fact that when comparing SB versus PPV for RRD repair there seems to be no difference on the final BCVA at the 1 year follow-up. It is very difficult to assess if this lack of difference was due to the study design, the way it was conducted, or because there is no real difference. For example, in the series published by Schaal et al., the final BCVA was equal among all the assessed techniques (22). However, the proportion of enrolled phakic and pseudophakic patients were also almost the same (54% vs. 46%) (22). Conversely, the series published by Wong et al. in 2014 clearly favored SB in terms of final BCVA, had almost 4 times more phakic than pseudophakic patients, and 61% of the population had a macula on status (36). Regarding the functional outcome, the SPR study reported that in phakic patients, chain formation, a high number of breaks, total retinal detachment, extension of the detachments central to the major vessels, and low baseline BCVA were associated with the worst final BCVA at the end of the follow-up (32). Other studies had pointed out the rate of recurrences and the length of the clinical symptoms prior to repair as possible predictive factors for final BCVA (3, 32).

Whenever SB is selected as the primary method of retinal detachment repair, special consideration should be given to the preoperative macular status regardless of the phakic/pseudophakic status (17). Patients with the macula intact during presentation seem to have greater rates of primary anatomical success and a better final BCVA (26, 37) Furthermore, macular recovery tends to be slower with SB since the evidence points toward a higher incidence of residual subretinal fluid (SRF), including submacular fluid that tends to correlate with worse or slower recovery of visual acuity when compared to PPV (28, 38, 39). The latter may have greater weight when treating patients with longer history of macular involvement. Finally, patients with a detached macula at the time of presentation are more prone to redetachment when treated with SB (OR 3.7, 95% CI: 1.06–13.45, P = 0.03) (37).

## Primary Pars Plana Vitrectomy for Uncomplicated Mid-Severity RRD

Most retinal surgeons will agree that PPV is the technique of choice when dealing with complicated cases of RRD (27, 28), such as the ones associated with high grade PVR, giant tears, choroidal detachment, or macular hole (25). The technical advancement in small-gauge instrumentation and wide-field viewing systems have expanded the indication of PPV for RRD, to cases of lesser severity where it was not considered previously (13, 17), arguably because there was a lower chance of missing a retinal break during surgery (3, 13, 14, 18, 26). Some studies have estimated that in cases where PPV is chosen as the primary reattachment technique, 98% of the lesions are found intraoperatively (40). Furthermore, when comparing the anatomical and functional outcomes between the different available vitrectomy probe gauges, there seems to be no difference at all (41-45). In addition, most of the studies agree it is a safe procedure, with high success rates and has a definite impact on final visual acuity (22, 37, 40, 43, 44). PPV, contrary to SB, works by directly eliminating the traction forces exerted over the retina (2, 4). It could also prevent tear reopening by eliminating the vitreous as well as by directly peeling off structures responsible for exerting traction over the retina, or some parts of the retina, like proliferative membranes or the internal limiting membrane (2, 4). The technique´s primary anatomical success ranges between 64% to 100% depending on the series (10, 13, 14). Table 2 summarizes some of the most relevant studies and their respective results regarding RRD and PPV. The SPR, in its multiple reports, addressed the role of PPV in treating RRD of medium difficulty.

RCT: Randomized clinical trial. RS: Retrospective study. RCS: Retrospective Case Series. SB: Scleral Buckle. PPV: Pars Plana Vitrectomy. RRD: Rhegmatogenous retinal detachment; SMF: Submacular fluid; SRF: Subretinal fluid; VA: Visual acuity; BCVA: best corrected visual acuity; RF: Risk Factors; IOP: Intraocular pressure; PseudoP: Pseudophakic; OCT: Optical Coherence tomography; PVR: Proliferative vitreoretinopathy; Mo: Month; PCL: Perfluorocarbon liquid; Meta: Meta-analysis.

**Table 2:** Summary of the most relevant data regarding RRD and its surgical management with PPV since 2009.

**Year**	**Author**	**Type of Study **	**Main Outcome**	**Number of Participants.**
2009	Martinez-Castillo et al.	RCT	PPV for RRD with unseen breaks.	Phakic: 11; Aphakic/PseudoP: 50.
			Significant improvement in final BCVA (*p<0.01*), Primary anatomical outcome of 98%. Breaks found intraoperatively in 98% of cases.	Total: 61.
2009	Von Fricken et al.	RCS	25GS vs 20GA for RRD repairment.	Phakic: 52; Aphakic/PseudoP: 73.
			Primary anatomical outcome 90.6 and 91.8% respectively. Significant improvement in final BCVA in both groups (*p<0.01*).	Total: 125.
			Inferior detachment increases the risk for redetachment.	
2009	Mura et al.	RCS	25GA vitrectomy for RRD management.	Phakic: 75; PseudoP: 56.
			High rate of primary reattachment and significant improvement in final BCVA.	Total: 131.
2010	Kunikata & Nishida	RCS	Visual outcomes & complications of 25GA vitrectomy for RRD.	Macula on: 39; Macula off: 45.
			Primary anatomical outcome: 85.2%. Significant improvement in final BCVA (*p<0.01*).	Total: 84.
			Macula off group had more intra o postoperative complications than macula on (*p<0.01*).	
2010	Kim et al.	RCS	Persistent submacular fluid after SB and PPV for macula off RRD.	Phakic: 14; PseudoP: 2.
			Less persistent SMF with PPV at 1mo (*p=0.006*); Better BCVA at 6 and 12mo with PPV but not at the end of the follow-up.	Total: 16.
2010	Mansouri et al.	RS	Assessment of initial treatment in recurrent retinal detachment	Phakic: 36; PseudoP: 52.
			PPV group required more secondary procedures and was more prone to need silicon oil than SB and PPV/SB for retinal reattachment.	Total: 88.
2011	Heussen et al.	RCT	Risk factors that may lead to reoperations: SPF report no. 4.	Phakic: 416; PseudoP: 265.
			Recurrence significant reduced in Pseudo P with PPV and increased in phakic (*p<0.01*).	Total: 681.
			Less reoperations in PseudoP with PPV. Other RF for reoperations in PseudoP: worst initial BCVA, YAG capsulotomy, retina	
			incarceration, visual field defects, size and number of breaks.	
2011	Kinori at al.	RCS	PPV with & without SB for primary RRD repairment.	Phakic: 134; PseudoP: 47.
			Primary anatomical outcome: 81.3%. Similar anatomical and functional outcome in both groups.	Total: 181.
2011	Mehta et al.	RS	PPV vs. PPV/SB for primary RRD repairment.	Phakic: 37; PseudoP: 48.
			Similar anatomical outcomes in both groups in PseudoP. Less risk of redetachment with PPV/SB in phakic.	Total: 85.
2011	Schaal et al.	RS	1-year anatomical and functional outcome of 4 different surgical techniques for RRD repairment.	Phakic: 204; PseudoP: 238
			No difference in primary anatomical outcome or final BCVA among the 4 techniques.	Total: 442.
2011	Albrieux et al.	RS	23GS vs 20GA for RRD repairment.	Phakic: 16; PseudoP: 54.
			Both groups had similar primary anatomical outcomes and significant improvement in visual acuity.	Total: 70.
2012	Yanyali et al.	RS	Primary 23GA PPV for RRD.	Phakic: 26; Aphakic/PseudoP: 23
			Primary anatomical outcome: 95.9%. Visual acuity improved in 83.7%.	Total: 49.
2012	Schneider et al.	RCS	PPV without adjuvants (no SB, 360°C laser or PCL) for RRD repairment.	Phakic: 40; PseudoP: 55.
			Primary anatomical outcome: 95.7%. Final BCVA of 20/40 or better in 77.4%. VRP: 3.2%.	Total: 95.
2013	Huang et al.	RCS	SB vs. PPV for macula off RRD assessed by OCT.	Total: 26.
			81.3% in SB group had SRF at 8 weeks of follow-up (*p<0.05*). 42.3% in PPB developed ERM. PPV better for macular recovery.	
2013	Dell'Omo et al.	RS	25GS vs 20GA for inferior RRD repairment.	Phakic: 52; Aphakic/PseudoP: 33.
			No difference in primary anatomical success and visual acuity improvement between groups.	Total: 85.
2013	Feltgen et al.	RCT	Risk assessment of anatomical outcome. SPR report no. 7	Phakic: 425; PseudoP: 265.
			No significant difference between SB and PPV regarding anatomical outcome.	Total: 690.
			**Phakic:** Anatomical outcome negatively associated with: Number and size of breaks (>1 clock hour) and intraoperative cryotherapy.	
			Positively associated with: breaks with irregular borders and SRF drainage.	
			**PseudoP:** Anatomical outcome negatively associated with: Number of breaks and previous YAG capsulotomy.	
			Positively associated with: intraoperative use of laser in PPV.	
2013	Goto et al.	RS	PPV for RRD anatomical success rate: superior vs inferior detachments.	Phakic: 74; PseudoP: 8
			Worst anatomical outcome with inferior breaks (*p<0.05; OR: 11.8*); even worst in patients with more than 2 weeks of symptoms.	Total: 82.
2013	Bernheim et al.	RCT	RRD in phakic and pseudophakic patients with high myopia.	Phakic: 151; PseudoP: 40.
			Better anatomical outcome in PseudoP (*P=0.05*); Worst base line BCVA (<20/400), greater axial length and PPV were negatively	Total: 96.
			associated with final BCVA.	
2013	Adelman et al.	RCT	Surgical outcomes for uncomplicated RRD: EVRS report no. 1.	Phakic: 1159; PseudoP: 1076.
			Lower failure rate in PseudoP with PPV than with SB and PPB/SB (*P=0.048*).	Total: 2235.
2013	Soni et al.	Meta	Surgical manage of RRD: meta-analysis of RCTs	7 studies: 6 RCT, 1 meeting
			Better final BCVA with SB in phakic; No difference in primary anatomical or final BCVA between SB and PPV in PseudoP/Aphakic.	abstract
			PPV had better secondary anatomical outcome in PseudoP/aphakic than SB. PPV increases the risk for cataract (OR: 4.22).	
2013	Figueroa et al.	RCS	Anatomical and functional outcome after 23GA PPV for RRD.	Phakic: 50; PseudoP: 83.
			Statistical significant improvement in final BCVA but no difference between phakic and PseudoP.	Total: 133.
2014	Storey & Kaiser.	RS	PPV vs. PPV/SB for primary RRD with high risk for PVR.	Phakic: 22; PseudoP/Aphakic: 7.
			Better primary anatomical outcome with PPB/SB than with PPV alone; especially in younger patients. There was no difference in	Total: 29.
			patients of 65 years or older. Final BCVA was similar in both groups at the endo of the follow-up.	
2014	Orlin & Chan.	RS	Primary PPV vs. PPV/SB for non-complex RRD.	Phakic: 17; PseudoP: 35.
			Primary anatomical outcome in 83%. No difference with inferior lesions, PseudoP and small gauge vitrectomy.	Total: 52
2014	Kobashi et al.	RS	Anatomical outcomes of SB for RRD and prognostic factors for primary anatomical success.	SB: 271 (Phakic: 260).
			Primary anatomical outcome: 96.3%; Inferior lesions and lens status had no effect over primary anatomical outcome	PPV: 271 (Phakic: 228).
				Total: 542.
2014	Wong et al.	RS	Surgical trends for RRD in an Asian tertiary eye center.	Phakic: 102; PseudoP: 87.
			Primary anatomical outcome was better with PPV/SB than with PPV alone (*p<0.01*) but the final anatomical success rate was similar.	Total: 189.
			Worst functional outcome with PPV (p<0.01). PVR and older age had a negative association with functional outcomes in Phakic and	
			PseudoP.	
2014	Lee et al.	RS	Failed PPV for RRD: 3 years of follow-up.	Total: 113.
			72.9% of redetachments were diagnosed at 2 months of follow-up; 95% at three months and 97.7% at 6 months.	
			Final functional outcome was better with reapplication after failure and worst in cases with PVR.	
				Total: 6380 cases

They concluded that PPV significantly reduces the rate of recurrence in pseudophakic patients while improving the primary anatomical outcome (hazard ratio [HR] = 0.556, 95%-CI [0.393; 0.787], P = 0.0009) and lowering the rate of retina-affecting secondary procedures, when compared to that of the SB group (27, 31, 32).

This same observation has been replicated constantly in several studies and meta-analyses; like the one published by Heimann in 2007 in which better anatomical success was achieved in pseudophakic patients with PPV (6). In a prospective study, Bernheim et al. reported a higher final anatomical success in pseudophakic patients with high myopia (46). Moreover, they also identified a low baseline BCVA and an increased longitudinal axis as predictive factors for low final visual acuity (46). Interestingly, in studies that report high primary and final reattachment rates while using PPV for RRD, but not when directly comparing it with other techniques, the analysis of the population enrolled are predominantly pseudophakic patients (13, 40, 46). Although the EVRS study did not find a difference in anatomical failure by the end of the study between SB and PPV when treating RRD in pseudophakic patients, they did observe a lower rate of redetachments or complications that required additional surgery after the initial procedure (24, 25). They even did better than patients treated with PPV/SB procedures (24).

On the analysis of preoperative clinical characteristics associated with a higher rate of reoperations, hence with a worse primary anatomical success in pseudophakic patients (regardless of the surgical technique); a multivariate logistic regression model of the SPR study identifies large breaks (HR = 1.611, 95%-CI [1.050; 2.472], P = 0.0290), the number of breaks (HR = 1.144, 95%-CI [1.086; 1.205], P < 0.0001), the number of detached quadrants (HR = 1.245, 95%-CI [1.011; 1.533], P = 0.0387), and symptomatic visual field defects at presentation (HR = 0.595, 95%-CI [0.357; 0.992], P = 0.0463) as possible negative predictive factors (27). The mean number of breaks in patients with primary anatomical success was 2 (standard deviation [SD] ± 2), while the number without anatomical success was 4 (SD ± 3) (31). The number of breaks and the extension of the detachment have also been identified by other authors as relevant preoperative factors associated with anatomical outcomes (3, 31, 32, 47-49).

Regarding the functional outcome in pseudophakic patients, the SPR identified the number of retinal breaks (DF = 1, F = 11.03, P = 0.0010) and inferior detachment, with breaks below the 4 and 8 o´clock position (DF = 1, F = 5.75, P = 0.0173) as negative predictive factors for final BCVA (32, 50). Moreover, inferior detachments with lesions below the 4 and 8 o´clock position seems to also have the worst anatomical outcome (32). This observation is somehow controversial, since other studies have struggled to replicate the results (51, 52). Nevertheless, there are some small case series and retrospective studies that have reported such an association. Von Fricken et al. reported a higher redetachment rate in inferior detachments when comparing 20 to 25 gauge vitrectomies for RRD (41). Kinori et al., while failing to demonstrate a difference between SB/PPV and PPV for RRD, had a worse anatomical result (80.9%, p = 0.74) with inferior detachments (51). In 2013, Goto et al. reported a significantly lower anatomical success rate with PPV and inferior breaks, which were even lower when the symptoms lasted more than 2 weeks preoperatively (80% versus 98%, P < 0.01) (53). In his study, the presence of inferior lesions was also associated with a higher rate of redetachment (53). The formation of cataracts during the follow-up after PPV is a well-known factor that might negatively affect the final BCVA. This complication is easily overlooked in clinical trials, especially if the patients do not have adequate follow-up or if the study´s design does not acknowledge it as a possible confounder (6, 27, 28, 32). The need of additional surgery to address the cataract may positively impact the rate of secondary procedures needed after RRD surgery (4, 27). Therefore, care should be taken when analyzing the results of cohorts that are predominantly phakic with a short follow-up period or the definition of secondary surgery or surgical success is too vague. The same goes for predominantly pseudophakic cohorts when studies do not consider capsular fibrosis as a possible final BCVA cofounder. Contrary to SB, macular status during the initial examination of the patient does not correlate with anatomical outcome (43, 45). However, PPV does promote a speedy macular recovery with a lower incidence of SRF, both clinically and by optical coherence tomography (OCT), immediately and up to 8 weeks after the procedure, which could mean a better final BCVA in theory, due to a faster macular recovery, as suggested by Kim et al. and Huang et al (28, 38). Kunikata and Nishida have noticed an increased rate of complications during and after surgery in macula-off patients who underwent PPV as well (54).

## Proposed Summarized Algorithm and Conclusions

Retinal detachment surgery is a common vitreoretinal procedure, with a good overall rate of success (4, 37). However, unlike the rest of the surgeries in ophthalmology, the term “success” is used very loosely (55). It can mean many different things depending on the author. For example, it can mean just anatomical success, disregarding final visual acuity, or short term success, disregarding what happen after longer follow-up or the existence of long-term complications, or it can mean anatomical and functional success as the result of a cluster of surgeries and procedures, taken all together as a group. Although, in the last decade, there have been many technological advances aimed at improving the safety and outcomes of retinal detachment surgeries (3, 18), the procedures are still flawed with shortcomings. About 40% of the patients will not attain reading capabilities despite a successful reattachment. Between 10% to 40% of the patients will need additional procedures to ensure long-term success of the primary surgery. Despite all efforts, 5% of the eyes will have permanent anatomical or visual disability (6). One of the areas where further improvement is possible is in the personalization of the surgical technique, tailoring it towards the preparatory clinical characteristics and morphology of the detachment of each patient. Therefore, the selection of the surgical technique should be based on the clearly identified prognostic factors, instead the personal preference of the surgeon. Whereas anatomical success is indeed very important, functional results are becoming a major point of attention. Any modification to the existing paradigm should strive to improve both final visual acuity and reading capabilities.

Based on the evidence presented in the previous paragraphs, the authors believe that, although there is no definitive proof pointing towards a clear collection of pre-surgical clinical characteristics that may help the surgeon personalize the surgical technique to each case, the results from the various studies described herein, in addition to the collective experience of the retina department of our hospital, provide a good starting point for the design of a decision-making algorithm that can be used to help improve the SSSR. We based our conclusions on the following premises. First, in RRD cases that are at poles of severity there is little doubt about which surgical technique is needed; therefore these algorithms only apply to RRD cases of mid severity (PVR less of grade B). Second, phakic patients with RRD tend to do better with SB. Third, pseudophakic patient tend to do better with PPV. Fourth, a combined procedure of PPV/SB has the potential of improving anatomical outcome while decreasing the risk of redetachment. Fifth, a shorter time to macular recovery may result in better visual acuity. Sixth, whenever a buckle is needed, a 360° SB is the surgical technique of choice among the authors. However, this only reflects a teaching trend and does not mean that 360° is superior to radial or segmental buckles. Seventh, small gauge PPV is the new norm. Finally, all retinal surgeons should strive to be equally proficient in both techniques and teaching programs should provide equal exposure to both techniques.

**Figure 1:**
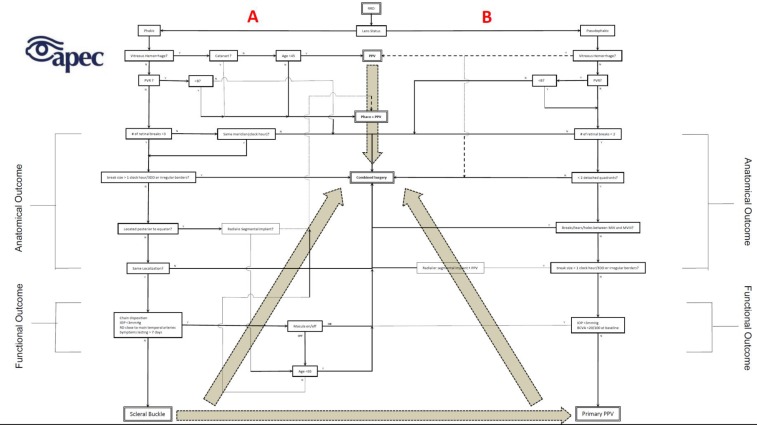
Graphic representation of the decision-making algorithm for the treatment of RRD in phakic patients and Pseudophakic patients. Side A should be followed in case of phakic patients at presentation. Side B should be followed in case of Pseudophakic patients at presentation. Solid lines with arrowheads are the critical pathway that must be followed in order to select the ideal surgical technique. The direction of the flow will depend solely on the clinical characteristics of the RRD at presentation. Dotted lines and squares are alternative pathways that the surgeons may choose without impacting the final anatomical or functional outcome. The broad dotted gray arrows in the back symbolizes the “surgeon confidence”; Which symbolizes that even with quality evidence pointing toward certain technique, the surgeon may end selecting another technique due to its lack of confidence, individual training or previous experiences. PVR: Proliferative vitreoretinopathy. <B: proliferative vitreoretinopathy grade B. PPV: Pars plana vitrectomy; SB: Scleral buckle; DD: Disk diameter; IOP: intraocular pressure; RD: Retinal detachment; Phaco: Phacoemulsification; Radial: radial or segmental buckle; BCVA: Best corrected visual acuity; #: number; M: meridian; Y: yes; N: No.

The evidence regarding combined PPV/SB procedures is not conclusive. Some studies support that the addition of a buckle improves the anatomical outcomes in special situation (13). There are plenty studies, such as the EVRS study where adding a buckle was not superior to PPV alone in decreasing the failure rate (24), that do not find any differences (36, 52). Nevertheless, the authors believe that in their hands, they have evidence that adding a buckle to PPV will improve the chances of favorable outcomes, while accelerating the visual recovery time by regaining attachment faster. According to premise #2, #6 and #8, the authors decided that the best way to treat phakic patients is with 360° SB (33). Figure 1 details the decision-making process for phakic patients.

Side A should be followed in case of phakic patients at presentation. Side B should be followed in case of Pseudophakic patients at presentation. Solid lines with arrowheads are the critical pathway that must be followed in order to select the ideal surgical technique. The direction of the flow will depend solely on the clinical characteristics of the RRD at presentation. Dotted lines and squares are alternative pathways that the surgeons may choose without impacting the final anatomical or functional outcome. The broad dotted gray arrows in the back symbolizes the “surgeon confidence”; Which symbolizes that even with quality evidence pointing toward certain technique, the surgeon may end selecting another technique due to its lack of confidence, individual training or previous experiences. PVR: Proliferative vitreoretinopathy. < B: proliferative vitreoretinopathy grade B. PPV: Pars plana vitrectomy; SB: Scleral buckle; DD: Disk diameter; IOP: intraocular pressure; RD: Retinal detachment; Phaco: Phacoemulsification; Radial: radial or segmental buckle; BCVA: Best corrected visual acuity; #: number; M: meridian; Y: yes; N: No. 

To improve the anatomical outcome, the authors selected the number of lesions, their subjective size, shape, and their anatomical situation as the most important predictive factors, based on the existing evidence. Although the best available evidence has identified 6±2 retina breaks as the critical number for anatomical failure (31). The authors’ experience dictates that trying to adequately treat more than three breaks simultaneously solely with a buckle increases the risk of failure due to the unintentional inadequate indentation of a lesion (especially if the lesions are located at different distances from the pars plana) while increasing the risk of PVR due to excessive cryotherapy (56). Another important anatomical predictive factor is the size of the largest retinal break, as well as, the shape of its borders. A lesion that is characterized as more than 3 disk diameters or 1 clock hour in size, as well as, rolled borders were considered to be more prone to anatomical failure (31, 32, 34, 35). As predictors for poor functional outcomes, the authors selected the chain disposition of several lesions, low intraocular pressure (< 3 mmHg) at baseline, detachments threatening the temporal arcades, and cases with symptoms lasting more than 7 days (32, 35). Since functional outcome is becoming more important nowadays, the authors believe that these factors should overrule the anatomical predictors, because having a reattached retina without improving the visual acuity of the patients should not be considered a complete success. Special consideration is needed whenever lesions posterior to the equator are present. Because, even in cases with a single, small lesion with regular borders, the surgeon can consider either placing a radial buckle/sponge or selecting PPV as primary reattachment technique. Finally, in cases where the functional outcome predictors were present, the surgeon should assess macular status as the next critical decision-making step. According to premises #4 and #5, in order to improve the functional and anatomical outcomes in cases of a detached macula, your primary concern should be a speedy macular recovery and ensuring that the retina will remain reattached (37). Therefore, a PPV/SB procedure is better suited for this scenario. In cases in where the macula is threatened but not completely detached, the age of the patient should be considered first. According to Storey et al., younger patients (< 65 years) tend to do better with PPV/SB procedures (57). However in older patients, PPV has the same outcomes than the PPV/SB (57). In this case, in order to avoid additional cataract surgery and decreased visual acuity in the future, a combined procedure of phacoemulsification and PPV is preferred due to the loss of accommodation in this age group.

According to premise #3, #7 and #8, the authors decided that the best way to treat pseudophakic patients is with small gauge PPV. Figure 1 details the decision-making process for pseudophakic patients. According to the existing evidence, the authors selected the number of lesions, their subjective size, shape, the inferior localization of the breaks (between 4-8 o´clock) and the number of detached quadrants as predictive factors for anatomical outcome (31, 32). In this scenario, the number of breaks associated with favorable anatomical outcome is 2±2 while a negative outcome is associated with 4±3 breaks (32). Therefore, the authors believe that the ideal number of breaks to safely treat a pseudophakic RRD should be zero to no more than three, taking into consideration that some pseudophakic RRD will not have any evident lesion during the initial fundus examination. In addition, more than 3 lesions will also mean a greater risk of PVR (50). Despite the fact that considering an inferior localization of a lesion as a negative predictive factor for anatomical outcome is controversial, the authors decided to include this as a critical point because in the case of PVR, having a preplaced buckle will improve the chances of success of a second surgery (58). The number of affected quadrants is also important not only because the SPR study associates it with worse anatomical outcomes, but the authors also agree that having more than 50% of the retina detached should be treated as a different surgical emergency that requires more aggressive approach (55). As predictive factors for poor functional outcome the authors selected low intraocular pressure (<3 mmHg) at baseline and BCVA of 20/100 or worse at the time of presentation (46, 55, 59). Similar to phakic patients, the authors believe that the presence of factors for poor functional outcome should overrule the importance of the anatomical predictive factors. Special consideration should be given to cases with a lesion larger than 1 clock hour/3DD or with irregular shaped borders. In those cases, depending on the surgeon’s experience, he may consider placing a segmental buckle instead of a full 360° buckle.

Both decision-making algorithms are based on evidence from peer review journals and the collective experience of more than 50 years of practice. In order to prove that these algorithms can improve the SSSR, a randomized controlled trial has been designed. Considering a one-tail alpha value, a 95% level of confidence with 80% power, and 10% of patient loss during the follow-up, we needed a total of 149 participants in order to prove an increase in the phakic group (SB SSSR 64%) with a delta of 16%. In the pseudophakic group (PPV SSSR 72%) with a delta of 15%, 137 participants are needed, with enough confidence the increase in the SSSR. For the PPB/SB group we decided to use the same data from the SB group and planned for 149 participants in this group as well. For the control group and because the SSSR is a very well-known outcome, we used a 2 to 1 proportion for a total of 218 participants. In total, for the validation of the algorithms, a total of 653 participants need to be randomized into the study and control groups. Due to its large size, a multicenter prospective randomized clinical trial design is needed with at least 10 participant centers in order to keep a reasonable enrollment phase of 1 year, with a follow-up phase of two years and review of the outcomes endpoints at 6, 12, 18 and 24 months.

Finally, the algorithms have some limitations that the authors would like to address. First, the decision-making process of each algorithm only takes into account clinical characteristics of the study eye without assessing other important factors of the fellow eye. The existence of factors such as previous history of retinal detachment in the fellow eye, previous surgical failure, history of trauma, organ loss and concomitant blinding diseases like diabetic retinopathy, age-related macular degeneration or glaucoma, could persuade the surgeon into choosing a more aggressive approach. Secondly, the algorithms do not take into consideration systemic or genetic diseases that could have additional impact on the anatomical outcome. Diseases like Marfan syndrome, Weill-Marchesani, Ehler-Danlos, and Stickler syndrome, among others, are well known to have a higher risk of poorer surgical outcomes and high probability of recurrences after RRD surgery (60, 61). Another important limitation is that the algorithm does not consider what we call the "surgeon uncertainty.” Not all surgeons are the same, not all of them have the same level of expertise, and more importantly, not all retina teaching programs are perfect. While the "ideal" program should strive to provide the same level of exposure to all surgical techniques, this is not always the case; surgeons without enough experience, in one or another technique, will probably tend to favor the surgical technique with which he/she is more comfortable or have better outcomes, regardless of clinical presentation. Finally, before any surgical situation, the patient must be informed about all possible surgical options available and their possible outcomes; including what would happen if no surgery is performed, complications, and unforeseen eventualities. With all this information, the patient could choose a different surgical plan than the one suggested by the algorithm.

In summary, RRD is a potentially blinding disorder that represents half of the surgical cases in vitreoretinal practices. Despite all the technological advances, its treatment seems to depend more on the surgeons’ preference than on verifiable clinical data. Based on evidence found in literature, in order to standardize the RRD treatment, improve the SSSR, as well as, the functional outcome, the authors proposed two critical decision-making algorithms along with the outline of a randomized clinical trial aimed to validate them.
